# Predicting Patterns of Distant Metastasis in Breast Cancer Patients following Local Regional Therapy Using Machine Learning

**DOI:** 10.3390/genes14091768

**Published:** 2023-09-07

**Authors:** Audrey Shiner, Alex Kiss, Khadijeh Saednia, Katarzyna J. Jerzak, Sonal Gandhi, Fang-I Lu, Urban Emmenegger, Lauren Fleshner, Andrew Lagree, Marie Angeli Alera, Mateusz Bielecki, Ethan Law, Brianna Law, Dylan Kam, Jonathan Klein, Christopher J. Pinard, Alex Shenfield, Ali Sadeghi-Naini, William T. Tran

**Affiliations:** 1 Department of Radiation Oncology, Sunnybrook Health Sciences Centre, Toronto, ON M4N 3M5, Canada; audrey.shiner@sri.utoronto.ca (A.S.);; 2 Biological Sciences Platform, Sunnybrook Research Institute, Toronto, ON M4N 3M5, Canada; 3 Institute of Medical Sciences, University of Toronto, Toronto, ON M5S 1A8, Canada; 4 Institute of Clinical Evaluative Sciences, Sunnybrook Health Sciences Centre, Toronto, ON M4N 3M5, Canada; 5 Department of Electrical Engineering and Computer Science, Lassonde School of Engineering, York University, Toronto, ON M3J 1P3, Canada; 6 Division of Medical Oncology, Department of Medicine, University of Toronto, Toronto, ON M5S 1A8, Canada; 7 Department of Anatomic Pathology, Sunnybrook Health Sciences Centre, Toronto, ON M4N 3M5, Canada; 8 Department of Radiation Oncology, Albert Einstein College of Medicine, New York, NY 10461, USA; 9 Department of Engineering and Mathematics, Sheffield Hallam University, Sheffield S1 1WB, UK; 10 Department of Radiation Oncology, University of Toronto, Toronto, ON M5S 1A8, Canada

**Keywords:** breast cancer metastasis, machine learning, prediction models, metastatic patterns

## Abstract

Up to 30% of breast cancer (BC) patients will develop distant metastases (DM), for which there is no cure. Here, statistical and machine learning (ML) models were developed to estimate the risk of site-specific DM following local-regional therapy. This retrospective study cohort included 175 patients diagnosed with invasive BC who later developed DM. Clinicopathological information was collected for analysis. Outcome variables were the first site of metastasis (brain, bone or visceral) and the time interval (months) to developing DM. Multivariate statistical analysis and ML-based multivariable gradient boosting machines identified factors associated with these outcomes. Machine learning models predicted the site of DM, demonstrating an area under the curve of 0.74, 0.75, and 0.73 for brain, bone and visceral sites, respectively. Overall, most patients (57%) developed bone metastases, with increased odds associated with estrogen receptor (ER) positivity. Human epidermal growth factor receptor-2 (HER2) positivity and non-anthracycline chemotherapy regimens were associated with a decreased risk of bone DM, while brain metastasis was associated with ER-negativity. Furthermore, non-anthracycline chemotherapy alone was a significant predictor of visceral metastasis. Here, clinicopathologic and treatment variables used in ML prediction models predict the first site of metastasis in BC. Further validation may guide focused patient-specific surveillance practices.

## 1. Introduction

As many as 30% of breast cancer (BC) patients will develop distant relapse following primary treatment, and this is dependent on the stage and BC subtype [1]. Despite significant progress in cancer therapies to improve cure rates and prolong survival, metastatic BC portends poor prognostic outcomes; the median survival interval is estimated as early as 18 months from the time of progression [1,2,3,4]. Several clinicopathological characteristics have been studied for their association with developing distant metastasis (DM) [5,6,7,8,9,10,11]. Patients with triple-negative BC (TNBC) exhibit a greater prevalence of DM with the earliest recurrences compared to those patients with human epidermal growth factor receptor-2 (HER-2)-enriched BCs [12]. In comparison, estrogen receptor-positive (ER+) BC demonstrates prolonged latency periods for DM. Previous studies have shown the most common first site of DM for ER+ BCs involves the bones, whereas ER-negative (ER−) BCs tend to recur more often in the viscera, particularly the brain and lungs [13]. Despite known factors for developing DM, there are limited models to predict the site of DM based on clinicopathologic and treatment information [14]

The addition of adjuvant radiotherapy to breast conservation or mastectomy has improved disease-free survival and local–regional recurrence risk in early-stage BC [15,16]. This is based on eliminating residual or microscopic disease following bulk excision of the primary lesion and involved lymph nodes. However, a fraction of patients will develop distant relapse even without local recurrence, and this mechanism is still poorly understood. Several biological processes have been proposed, including linear, parallel and branching models of metastatic dissemination [17]. Linear progression models describe a stepwise pattern, whereas recent data favor parallel dissemination that confers early and immediate changes in the tumor microenvironment, liberating tumor cells into circulation [17]. The branched model describes tumor cell genomic alterations supporting distant colonization [17]. In BC, metastatic lineage can be traced to the translocation of freed tumor cells into lymphatic or circulatory vessels that evade detection [18]. Currently, the initialized timing and likelihood of DM at a specific site remain unknown, and there is a need to better characterize metastatic BC patterns in the clinic.

Machine learning (ML) in oncology has undergone tremendous growth, potentially allowing for personalized care in BC [19,20]. ML in conjunction with a better understanding of clinicopathological risk factors and current breast cancer therapies have the potential to identify patients that require more intense clinical monitoring and further establish individualized surveillance guidelines, including adaptive follow-up imaging. Here, we report an analysis of BC clinicopathological characteristics using current systemic therapy and radiation treatment regimens associated with the time to and first single site of DM using statistical and ML methods.

## 2. Materials and Methods

### 2.1. Cohort and Dataset

The institutional research ethics board approved this study. This retrospective study evaluated 416 BC cases at a single institution between February 2007 to August 2017. This study aimed to evaluate the factors associated with the first site of DM; thus, all patients included for analysis developed DM following standard treatments during a 10-year follow-up interval period.

**Figure 1:**
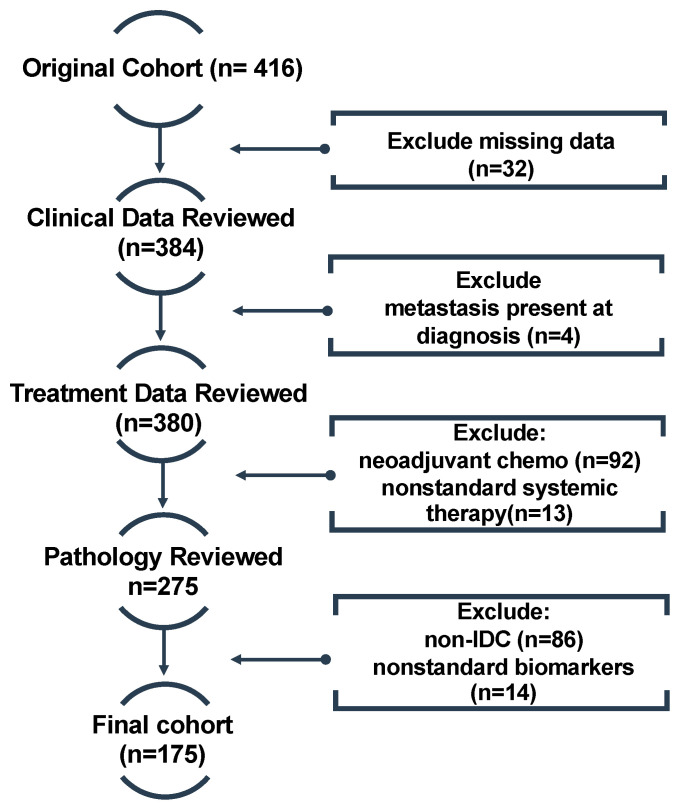
Flow chart displaying patient inclusion/exclusion criteria. Once patients with missing data were removed from the original cohort (n = 416), the remaining patients were excluded in order of clinical, treatment and pathological data. The final cohort for our study consisted of 175 patients. Abbreviations: chemo—chemotherapy; IDC—intraductal carcinoma.

Patient cases were screened for inclusion in the study using the institutional electronic medical records (EMRs) system. All patients received upfront surgery and postoperative radiotherapy. Selected patients received adjuvant systemic treatments according to the discretion of the treating medical oncologist. Both male and female patients were included in the initial data extraction. However, since male BC constitutes only 1% of all diagnoses, there were limited case numbers with metastatic progression and these were subsequently excluded from the final analysis. The age at diagnosis was retrieved for all women; patients between 18 to 80 years of age were included in the study to permit follow-up after treatment to diagnose DM. Once all exclusion criteria were implemented, the remaining analysis cohort comprised 175 patients (Figure 1).

Distant metastasis was radiologically confirmed under standard imaging protocols using computed tomography (CT) of the chest, abdomen, pelvis and head, or magnetic resonance imaging (MRI). Osseous metastatic involvement was also diagnosed using conventional Tc-99 m scintigraphy (bone scan). Only patients with a single metastatic site (i.e., bone, visceral or cranial lesion) at the time of progression were included for analysis (i.e., synchronous metastatic diseases were excluded). Those with locally recurrent disease at the time of metastasis were also excluded. This approach was implemented to meet the primary endpoint of modeling the first site of distant metastases using statistical and ML measures. Medical imaging assessments for the presence of DM were performed and reported by a board-certified radiologist. All data were extracted from EMRs.

### 2.2. Clinicopathological Variables

Diagnostic information on the primary BC was collected, including laterality, Nottingham grade, biomarker status and histological type. Only patients with invasive ductal carcinoma (IDC) were included for analysis. Other histological types, such as invasive lobular carcinoma, were excluded due to variances in metastatic patterns and treatment response profiles [21]. Patients who also presented with de novo metastatic disease or developed distant relapse during adjuvant therapy were excluded. A board-certified pathologist specializing in BC completed all pathologic reviews.

Synoptic pathology information was collected from surgical specimens for each patient. Final pathologic characteristics included type of surgery (lumpectomy vs. mastectomy), tumor size, nodal status, Nottingham grade (G1-3), lymphovascular invasion (LVI) and receptor status (ER, progesterone receptor (PR), and HER2). Staging information was captured for each patient according to the American Joint Cancer Committee (AJCC), eighth edition [22]. ER, PR and HER2 receptor status was assessed using immunohistochemistry (IHC) in accordance with the American Society of Clinical Oncology (ASCO) and College of American Pathologists (CAP) guidelines [23,24]. HER2-equivocal (2+) tumors were evaluated with fluorescence in situ hybridization assay (FISH).

The proliferative marker Ki-67 was not assessed as this was not part of the clinical care standard within the study period. Thus, the receptor status and tumor grade were used to define the following BC subtypes based on previous methods [25]: specifically, luminal A-like (ER+, PR+, HER2−), luminal B-like (ER+, PR+/−, HER2+ or HER2-negative but G3), triple-negative (ER-, PR-, HER2-), or HER2-enriched (ER-, PR-, HER2+).

### 2.3. Treatment Characteristics

All included patients underwent surgery and adjuvant radiotherapy. Patients who underwent breast-conserving surgery (BCS) with pathologic node-negative disease received whole-breast radiation (50 Gy/25 fx or 4256 Gy/16 fx or 40 Gy/15 fx) using a standard tangential field technique. Those with node-positive disease at the time of surgery underwent local–regional radiation, i.e., breast and nodal fields, including the internal mammary chain nodes, at the discretion of the treating radiation oncologist. Post-mastectomy radiation (PMRT) was administered to patients with high-risk characteristics (e.g., triple-negative phenotype, LVI-positive, or positive tumor margins) and those who demonstrated pathologic T3 tumors (chest wall only) or node-positive disease (local, regional treatment).

Boost treatments (breast or chest wall radiotherapy) were administered based on close (<1 mm) or positive resection margins, patients who were ≤50 years old, or those patients with several high-risk clinicopathologic features for local recurrence (e.g., triple-negative phenotype, ≤40 years of age, close or positive margins). Radiation treatments were administered using 3D-conformal forward-planning techniques, or intensity-modulated radiotherapy.

Adjuvant systemic therapy characteristics encompassed chemotherapy, endocrine therapy and targeted therapy (e.g., trastuzumab for HER2+ BC). Chemotherapy regimens were grouped as follows: anthracycline backbone alone (adriamycin [generic name: doxorubicin]) and cyclophosphamide [AC]; 5-fluorouracil, epirubicin, cyclophosphamide [FEC]); anthracycline–taxane backbone (AC followed by docetaxel [ACD]); AC followed by taxol ([generic name: paclitaxel]) [ACT]; FEC followed by docetaxel [FECD]; FEC followed by paclitaxel [FECT]; other (paclitaxel, docetaxel, capecitabine and docetaxel & cyclophosphamide [TC]); unknown; or none. Moreover, endocrine therapies for patients with hormone-positive BC included aromatase inhibitors and selective estrogen receptor modulators (SERMs), unknown or none. Any patients who received nonstandard drug therapy, neoadjuvant therapy or were involved in a clinical trial were excluded.

#### 2.3.1. Clinical Endpoints

The outcome measures of the study were the first single site of distant relapse and time to DM for each patient. Sites of metastases were classified as bone, brain and visceral. Visceral disease included lung, liver, and organs of the mediastinum. Time to DM was determined as the time (months) between initiating radiation therapy for the primary BC and the diagnosis of DM.

#### 2.3.2. Statistical Analyses

Descriptive statistics were calculated for all variables. Continuous measures were summarized using the mean, median and standard deviations, whereas categorical measures were summarized by frequency and percentages. The frequency and proportion of patients were calculated according to clinicopathological characteristics in an overall analysis and by outcome variable (Table 1, Table 2 and Table 3).

The classification outcome of the distant metastatic site (skeletal, brain, visceral) was analyzed using logistic regression models. For skeletal and brain outcomes, bivariate and multivariable modeling was carried out. For visceral metastases, a bivariate model was developed given the limited number of cases, which did not allow for multivariable modeling. Results of the logistic regression models were presented as odds ratios (OR) and their associated 95% confidence intervals.

Prior to multivariable model development, the set of predictor variables of interest was assessed for the presence of multicollinearity using tolerance statistics. A tolerance value of <0.4 was used as the cut-off point to detect the presence of multicollinearity. In such cases, only one member of a correlated set was retained for the multivariable model.

Poisson regression models were used to analyze the association between the clinicopathological characteristics and the number of days to metastasis. Results were presented as incidence rate ratios and their associated 95% confidence intervals. All analyses were conducted using SAS software Version 9.4 (SAS Institute, Cary, NC, USA) [26].

#### 2.3.3. Machine Learning Classifiers

Several supervised ML models were considered for this study, including naive Bayes, support vector machines (SVMs), k-nearest neighbors (K-NN), and gradient boosting machines (GBM). Each model possesses its advantages and disadvantages in handling medical data. For example, the naive Bayes is computationally fast and comprises simple hyperparameter tuning but is limited in the assumption of independence between attributes, which may reduce the predictive performance [27]. SVMs work well with linear and nonlinear datasets but struggle with overlapping or noisy datasets that affect the accuracy of building the hyperplane (decision boundary) [27,28]. K-NN algorithms are useful in handling missing data within a sample. However, model performances are reduced with highly dimensional datasets. K-NN algorithms also assume that the attributes are equally weighted in importance, which may result in inaccurate estimates of the predicted outcome [28].

Among the various ML algorithms, GBMs are attractive due to their versatility in approaching classification or regression problems and handling both parametric and nonparametric datasets. GBMs constitute an ensemble approach, using decision trees and building upon weaker models to enhance the ensemble prediction [29,30]. A sequential process of adding new weak learners at each iteration (i.e., boosting) is dependent on a loss function and ultimately yields a more robust prediction estimate. Thus, GBMs are highly flexible and customizable, attributing to their strengths to carry out prediction tasks. Newer GBM models, such as the XGBoost, replace the sequential framework with a multi-threaded approach, which enhances the computational speed to output a predicted outcome variable. With these considerations, we approached our analysis using an XGBoost classifier based on its flexibility to handle the various data types within the study cohort, the computational speed of the algorithm, and high potential of accurate predictions from the input data frame.

Three gradient boosting machines (GBMs) with decision tree models were used to predict the site of DM. The final ML models were developed and trained in the Python programming language (3.8.10) using the Scikit-learn (0.23.2) and XGBoost libraries (1.4.0) [31]. Data were partitioned on the patient level into a training set (75%) and an independent test set (25%) using a stratified K-fold approach [32]. Accordingly, 131 patients were used for the training and validation of the models, and 44 patients were used as an unseen test set for internal model validation. The missing values for LVI status (n = 14) and Nottingham grade (n = 1) were imputed using the mode values of the training set. Continuous features were scaled to zero and one using a min–max scaler before analysis. Scaling parameters were calculated based on the values in the training set and applied to all samples. The GBMs with decision trees were trained separately as a DM predictor for each site. A five-fold cross-validation on the training set was used with hyperparameter tuning. The hyperparameters were tuned as: (i) maximum depth of trees {‘max_depth’: 4}, (ii) maximum number of boosting trees {‘n_estimators’: 500}, (iii) learning rate {‘eta’: 0.1}, (iv) L1 regularization term on weights {‘alpha’: 0.02}, (v) subsample ratio of the training instances {‘subsample’: 0.9}, and (vi) negative-to-positive class ratio {‘scale_pos_weight’: 0.77}. The contribution of each feature to the prediction model was calculated based on its importance gain score. The features with the most significant contribution to the model that showed a meaningful difference in importance gain score compared to the rest of the features were identified in each cross-validation fold. A majority voting strategy was used to select the features with the highest contribution to the prediction models, i.e., the selected features in the optimal feature set were the ones identified in three or more folds out of five. A class weighting strategy was utilized in the GBM models to address data imbalances in the feature set [30]. The final prediction model for each DM site was trained with an associated optimal feature set on the entire training set and evaluated on the independent test set using accuracy, sensitivity, specificity and area under the receiver operating characteristic (ROC) curve (AUC). A threshold value of 0.5 was used as the cut-off to calculate sensitivity and specificity.

## 3. Results

### 3.1. Clinicopathological Characteristics

The clinicopathological characteristics of patients are presented in Table 1. The mean age at diagnosis was 55.6 ± 13.4 years, with 66 patients (38%) under the age of 50 years. The average tumor size was 31.5 ± 18.8 mm, with the largest proportion (n = 106 patients; 61%) of patients with pathologic T2 tumors. All patients had unifocal primary lesions. Nodal status varied, with 30%, 37%, 21% and 13% of patients presenting with N0, N1, N2 and N3 involvement, respectively. As for receptor status, 112 patients were ER+, 109 were PR+ and 34 were HER2+. Luminal A BC constituted the largest proportion of patients (n = 99 patients; 57%). There were 20 patients with luminal B subtypes, 14 with HER2-enriched tumors and 49 women who presented with TNBC. Moreover, 77% of the cohort received chemotherapy, 59% of patients underwent endocrine therapy and 23% were treated with anti-HER2-targeted therapy, trastuzumab. This cohort did not include other targeted agents used, such as pertuzumab (HER2+), or immunotherapies, including pembrolizumab.

The median follow-up period for all patients was 35 ± 29 months. Two outcomes for subsequent classification were measured: (1) the first site of DM and (2) the time interval between initial diagnosis and distant relapse (Table 2). The clinicopathological characteristics of patients according to clinical outcome measures are presented in supplementary Tables S1 and S2. There were 99 patients (57%) with bone DM, 55 women (31%) who developed brain DM and 21 (12%) cases identified with visceral DM. In addition, 22 patients (13%) recurred at or before one year post diagnosis (after completing primary treatments), 40 (23%) in the second year, 33 (19%) in the third year, 21 (12%) in the fourth year, 22 (13%) in the fifth year and 37 (21%) after more than five years. Furthermore, the distribution of metastatic sites over time, the average time of metastasis to each site and site breakdown according to BC subtype are displayed in supplementary Tables S3–S5.

### 3.2. Outcome Measures

#### 3.2.1. First Site of Distant Metastasis

**Figure 2:**
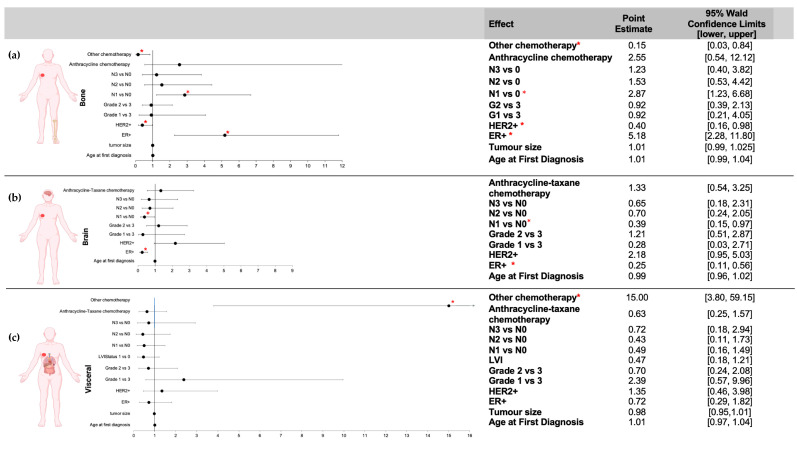
Odds ratios (OR) for bone, brain or visceral metastases according to clinicopathological characteristics. Multivariate analyses were conducted to determine the ORs for developing bone (**a**) or brain (**b**) metastasis according to clinicopathological characteristics. ER+ and N1 vs. N0 stage were significantly associated with an increased risk of bone metastasis, whereas HER2+ and “other chemo” were significantly associated with a decreased risk. ER+ was significantly associated with a decreased risk of brain metastasis. (**c**) ORs for developing visceral metastasis were analyzed using bivariate analysis, and no characteristics were significant. Abbreviations: chemo—chemotherapy; N0—nodal status 0 (0 positive nodes); N1—nodal status 1 (1–3 positive nodes); N2—nodal status 2 (4–9 positive nodes); N3—nodal status 3 (greater than 10 positive nodes); HER2—human epidermal growth factor 2; ER+—estrogen receptor-positive. (* indicates statistically significant, *p* = 0.05).

Odds ratio estimates for the association of clinicopathological characteristics with bone, brain or visceral metastasis are shown in Figure 2. In multivariate analysis, the odds of the first DM site being bone metastasis were significantly increased by ER positivity (*p* < 0.0001; OR = 5.2, 95% CI 2.3–11.8) and N1 stage compared to N0 (*p* = 0.05, OR = 3.0, 95% CI 1.4–6.4), as well as significantly decreased for patients positive for HER2 irrespective of ER status (*p* = 0.04; OR = 0.4, 95% CI 0.2–0.98) and those that underwent the group of “other” chemotherapy regimens, including paclitaxel, docetaxel and TC (*p* = 0.03; OR= 0.15, 95% CI 0.27–0.84). In contrast, ER+ patients had significantly lower odds of brain DM than ER- (*p* = 0.0009; OR = 0.2, 95% CI 0.1–0.6), as did patients with N1 compared to N0 status (*p* = 0.03, OR= 0.4, 95% CI 0.15–0.97). In bivariate analysis, the chemotherapy regimens grouped as “other” were the only significant predictor of visceral first DM site (*p* = 0.0001; OR = 15.0, 95% CI 3.8–59.1).

#### 3.2.2. Time to Distant Metastasis

In a multivariate analysis using a Poisson regression model, all variables tested were significantly associated with time to DM (Table 3). Each unit increase in age or tumor size, as well as increased nodal stage (N) and Nottingham grade (G), demonstrated a decrease in days to DM. In contrast, both ER+ and HER2+ patients showed greater latency periods to DM compared to those with ER- and HER2- disease, respectively. Furthermore, the use of anthracycline and anthracycline–taxane chemotherapies prolonged the time to metastasis.

### 3.3. Machine Learning Classification

The optimal feature sets and performance of the ML models developed for different DM sites are presented in Table 4. The accuracies of prediction models on the training and independent test sets range from 72% to 75% and from 70% to 75%, respectively. The test sensitivity and specificity of the models are within the ranges of 60%–72% and 68–77%, respectively. The ROC curves obtained for the three models on the independent test set are shown in Table 4. The test AUCs of the models were 0.75, 0.74 and 0.73 for predicting DM in the skeletal, brain and visceral sites, respectively.

## 4. Discussion

This study provides an analysis of clinicopathological and treatment characteristics associated with the first site of DM and time interval in early-stage BC following local–regional treatment. In correspondence to previous works, characteristics associated with a greater risk of metastasis overall include increased nodal stage, tumor size, Nottingham grade and presence of LVI [5,6,7,8,33]. Molecular and intrinsic subtypes, such as TNBC and HER2-enriched BCs, have also been shown to confer higher rates of distant relapse than luminal-type BCs [34]. Three separate GBMs, each comprised of several decision trees were developed in our study to predict each site of DM (bone, brain, visceral). GBMs were selected as classifiers due to their tendency to outperform random forests or ensemble models, as at each step the tree is trained to correct existing errors, enabling the model to capture more complex patterns [35]. Receptor status remained a significant predictor of the specific site of DM in our study, which agrees with previous studies; specifically, ER+ and HER2- BCs were significantly associated with an increased risk of bone as the first site of DM, whereas ER- BC was significantly associated with brain metastasis [13,36,37,38]. Interestingly, 71% of patients with visceral metastasis as their first DM site were under 50 years old in our study. In alignment with these findings, Frank et al. (2020) found that younger patients had a higher propensity for visceral DM [5].

Adjuvant systemic treatment was associated with variable outcomes. Patients treated with non-anthracycline-containing chemotherapy, including paclitaxel, docetaxel, capecitabine and TC, were significantly associated with decreased odds of bone metastasis. In contrast, there was an increased odds for visceral metastasis and time to DM. Other factors associated with an earlier interval to DM include node positivity, increased tumor size and grade; these findings align with a study by Colzani et al. (2014) [36]. Other studies have shown contrasting outcomes related to a decrease in time to metastasis per unit increase in age; specifically, younger patients had a greater risk of developing metastasis sooner than older patients [36]. Moreover, we found that ER+ and HER2+ increased the time to metastasis, whereas ER-negative BCs tended to metastasize earlier. This is supported by previous findings that indicate a heightened risk of metastasis for ER- patients within the first two years after diagnosis, compared to ER+ patients, who have an increased risk later on [36,38].

Previous studies have aimed to train ML models to predict the likelihood of survival of BC patients [39,40,41,42]. More recently, however, there has been an increasing interest in predicting the risk of metastasis as well, as it is a hallmark of ultimately fatal disease progression. For example, Song (2021) conducted a study using image-based features of BC tumors obtained from positron emission tomography/computed tomography (PET/CT) to predict the risk of axillary lymph node metastases in patients diagnosed with IDC [43]. Moreover, Tapak et al. (2019) compared the performance of various ML techniques to predict the risk of metastasis in BC patients. Similar to our study, they used clinicopathological characteristics as predictor variables for DM, including age, grade, stage, receptor statuses and different surgical approaches [44]. However, the outcome variable was generalized as the overall risk of DM occurrence. Our study is novel as we aimed to identify both the site- and time-specific risks of DM, which can potentially guide more focused surveillance and screening for at-risk patients. Current ASCO guidelines encourage clinicians to individualize clinical follow-up for their patients. Asymptomatic low-risk BC patients may undergo a modified surveillance program involving less frequent screening intervals [45]. This may hinder the early detection of metastatic spread or prevention of metastasis for each patient, as metastases often remain undetected until symptomatic or in circumstances where organ function is affected. This often presents in late-stage organ invasion and yields poorer prognostic endpoints [46,47]. Despite the need to individualize follow-up care, clinical decision support tools to guide practices are limited. Due to the lack of validated assays, metastatic onset remains elusive in the oncology clinic. However, several reports have yielded regression-based nomograms [14,48,49,50,51,52]. For example, Ye and colleagues [52] used data from the Surveillance, Epidemiology, and End Results (SEER) program to build an LR-based nomogram to predict bone metastases only in BC patients. Clinical factors included age, grade, histologic type, surgery of breast lesions and BC subtypes. The model’s performance corresponded with an AUC = 0.689 from the internal validation set. Similar to our study, Lim et al. evaluated the risk of distant failure in BC patients following radiotherapy. Their nomogram accounted for clinicopathologic variables associated with metastatic relapse, and all distal sites were grouped together in the analysis. The prognostic model was built from a Cox regression model with a concordance index of 0.812 [49]. There is no current clinician-based “gold-standard” to compare if these models outperform routine clinical judgement, but they demonstrate promise in the development of a practical patient decision support tool.

Existing clinical decision support tools are used to predict the risk of BC recurrence and the putative benefit of adjuvant systemic treatment. A substantial body of work has focused on exploiting genomic signatures, yielding assays such as Oncotype DX (Genomic Health, Redwood City, CA, USA) [53], Mammaprint (Agendia BV, Amsterdam, The Netherlands) [54], EndoPredict (Myriad Genetics Inc., Salt Lake City, UT, USA) [55] and PAM50/Prosigna (NanoString Technologies, Seattle, WA, USA) [56]. The selection of candidate genes was based on hormone receptor expression, HER2 signaling, proliferative markers and clinical validation in patients with a low nodal burden [53,54,55,56]. These assays are limited to ER+ patients, who have distinct metastatic and relapse patterns compared to triple-negative and HER2-positive BCs.

Risk stratification according to the specific site of DM may enable disease-specific surveillance practices and treatments. This could involve modifications in the frequency of surveillance imaging, ascertain the indication for additional diagnostic tests and enhance future research in the early prediction of DM through serum markers. There is a growing interest in circulating tumor cells (CTCs) as a measure of metastatic risk in BC. Published data report that elevated CTCs in the bloodstream are associated with an increased risk of DM and, therefore, a poorer prognosis [47,57,58]. In addition, previous studies reported that elevated concentrations of serum biomarkers, including cancer antigen 15-3, carcinoembryonic antigen and cancer antigen 125, are associated with DM [34,59]. Future statistical and ML models could potentially guide routine CTC and serum biomarkers for specified high-risk groups and enable earlier detection of DM [60,61].

The limitations of this study include a small number of subjects and grouping “visceral DM” from several subsites. The patient cohort was derived from a single institution, which limits the generalizability of our findings and would benefit from an external validation cohort. Furthermore, time to DM was measured as the time elapsed between the initiation dates of radiation treatment for the primary BC and diagnosis of DM.

## 5. Conclusions

Identifying which BC patients are at higher risk of DM and, more specifically, the sites and time points of interest is critical for stopping its spread early and possible prevention of DM altogether. While validation is needed, and the limitations of this study must be addressed, our promising findings and predictive models proposed can serve as a basis to guide future research.

## Figures and Tables

**Table 1:** Clinicopathological characteristics including pre-surgical characteristics, surgical pathology and adjuvant treatments. Chemotherapy treatments were grouped as follows: anthracycline backbone alone (AC, FEC, FEC100), anthracycline–taxane backbone (ACD, ACT, FECD, FECT), other (paclitaxel, docetaxel, capecitabine, TC) and unknown. Outcome variables of DM included the site of DM and time to DM. Abbreviations: T stage—tumor size; N stage—nodal status; M stage—metastasis; G1—Nottingham grade 1; G2—Nottingham grade 2; G3—Nottingham grade 3; SD—standard deviation; ER+—estrogen receptor-positive; PR+—progesterone receptor-positive; HER2+—human epidermal growth factor-positive; LVI—lymphovascular invasion; NA—not available; TNBC—triple-negative breast cancer.

Clinicopathological Characteristics	Study Cohort (n = 175)
**Pre-surgical Characteristics**	
Age	
Mean Age ± SD (years)	55.6 ± 13.4
20–49 years	66 (38%)
≥50 years	109 (62%)
Laterality	
Left	93 (53%)
Right	82 (47%)
**Surgical Pathology Characteristics**	
Type of surgery	
Lumpectomy	122 (69%)
Mastectomy	53 (30%)
T Stage	
Mean size ± SD (mm)	31.52 ± 18.80
N Stage	
N0	52 (30%)
N1	64 (37%)
N2	36 (21%)
N3	23 (13%)
Nottingham Grade	
G1	12 (7%)
G2	56 (32%)
G3	106 (61%)
NA	1 (1%)
Receptor Status	
ER+	112 (64%)
PR+	109 (62%)
HER2+	34 (20%)
Subtype	
Luminal A	99 (57%)
Luminal B	20 (11%)
HER2-Enriched	14 (8%)
TNBC	49 (28%)
LVI Status	
LVI-	69 (39%)
LVI+	92 (53%)
NA	14 (8%)
**Adjuvant Treatments**	
Chemotherapy (n = 135 (77%))	
Anthracycline backbone alone	9 (5%)
Anthracycline–taxane backbone	101 (58%)
Other	10 (6%)
Unknown	15 (9%)
Endocrine Therapy (n = 103 (59%))	
Aromatase Inhibitors	43 (25%)
Tamoxifen	47 (27%)
Unknown	13 (7%)
Trastuzumab	23 (13%)

**Table 2:** Outcome variables: the site of distant metastasis, and time to distant metastasis, are shown according to population breakdown. Sites of distant metastasis were classified as bone, brain and visceral (lung, liver, organs of the mediastinum) and time is presented in years.

Outcome Variables	Study Cohort (n = 175)
**Sites of Distant Metastasis**	
Bone Metastasis	99 (57%)
Brain Metastasis	55 (31%)
Visceral Metastasis	21 (12%)
**Time to Distant Metastasis**	
≤1 year	22 (13%)
>1–≤2 years	40 (23%)
>2–≤3 years	33 (19%)
>3–≤4 years	21 (12%)
>4–≤5 years	22 (13%)
>5 years	37 (21%)

**Table 3:** Analysis of the outcome of days to first distant metastasis. A multivariate analysis was conducted using Poisson regression models. All clinicopathological variables shown were significantly associated with time to distant metastasis (α = 0.05). Abbreviations: CI—confidence intervals; ER+—estrogen receptor-positive; HER2—human epidermal growth factor 2; G1—Nottingham grade 1; G2—Nottingham grade 2; G3—Nottingham grade 3; LVI—lymphovascular invasion; chemo—chemotherapy; N0—nodal status 0 (0 positive nodes); N1—nodal status 1 (1–3); N2—nodal status 2 (4–9); N3—nodal status 3 (>10).

Contrast Estimate Results			
Label	Incidence Rate Ratio	95% CI	*p*-Value
Age	0.99	[0.99, 0.99]	<0.0001
Tumor size	0.99	[0.99, 0.99]	<0.0001
ER+	1.98	[1.96, 2.01]	<0.0001
HER2+	1.14	[1.12, 1.15]	<0.0001
Grade 1 vs. 3	1.15	[1.13, 1.17]	<0.0001
Grade 2 vs. 3	1.01	[1.00, 1.02]	0.0378
LVI	1.05	[1.04, 1.06]	<0.0001
N1 vs. 0	0.69	[0.68, 0.69]	<0.0001
N2 vs. 0	0.86	[0.84, 0.87]	<0.0001
N3 vs. 0	0.71	[0.70, 0.73]	<0.0001
Anthracycline–taxane-based	1.57	[1.54, 1.60]	<0.0001
Anthracycline-based	1.15	[1.14, 1.17]	<0.0001
Other chemo	0.83	[0.81, 0.84]	<0.0001

**Table 4:** Results of distant metastasis prediction at different sites using clinicopathological features on the training and test sets. The ROC curves of the test set for each corresponding site are displayed. The features included in each optimal biomarker are listed. Abbreviations: Acc—accuracy; AUC—area under the curve; Sen—sensitivity; Spec—specificity; Tr—training; Val—validation; Te—test; ER+—estrogen receptor-positive; PR+—progesterone receptor-positive; HER2+—human epidermal growth factor 2-positive; T stage—stage of tumor size; N stage—stage of nodal status; ROC—receiver operating characteristic.

	Visceral	Brain	Skeletal
**Selected features**	- ER status - HER2 status - LVI status - Adjuvant tamoxifen - Aromatase inhibitors - Adjuvant chemo - Anthracycline–taxane backbone (A.C.D., A.C.T., FECD, FECT)	- PR status - HER2 status - LVI status - Mastectomy - Adjuvant trastuzumab - Aromatase inhibitors - Adjuvant chemo - OTHER chemo (paclitaxel, docetaxel, capecitabine, TC)	- ER status - LVI status - Lumpectomy - T stage - N stage - Nottingham grade - Adjuvant trastuzumab - Aromatase inhibitors
**Tr Acc**	0.72	0.75	0.73
**Tr Sens**	0.64	0.73	0.75
**Tr Spec**	0.73	0.78	0.72
**Te Acc**	0.70	0.75	0.70
**Te Sens**	0.60	0.71	0.72
**Te Spec**	0.72	0.77	0.68
**Te AUC**	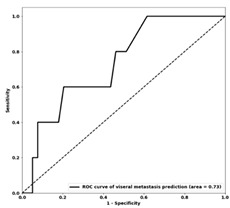 0.73	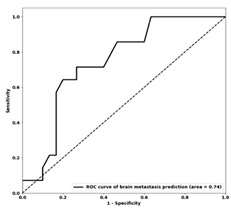 0.74	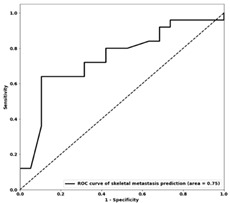 0.75

## Data Availability

The dataset curated and analyzed during the current study is not available to the public, however, may be made available by the corresponding author upon reasonable request.

## Supplementary Materials

The following supporting information can be downloaded at: https://www.mdpi.com/article/10.3390/genes14091768/s1, Table S1: Clinicopathological characteristics of patient cohorts grouped by site of first distant metastasis. Sites of distant metastasis were classified as bone, brain and visceral (lung, liver, organs of the mediastinum). Chemotherapy treatment was grouped as follows: anthracycline backbone alone (AC, FEC, FEC100), anthracycline–taxane backbone (ACD, ACT, FECD, FECT), other (paclitaxel, docetaxel, capecitabine, TC) and unknown. Abbreviations: T stage—tumour size; N stage—nodal status; M stage—metastasis; G1—Nottingham grade 1; G2—Nottingham grade 2; G3—Nottingham grade 3; SD—standard deviation; ER+—estrogen receptor-positive; PR+—progesterone receptor-positive; HER2+—human epidermal growth factor 2-positive; TNBC—triple-negative breast cancer; LVI—lymphovascular invasion; NA—not available. Table S2: Clinicopathological characteristics of patient cohorts grouped by time in years until first distant metastasis. Chemotherapy treatment was grouped as follows: anthracycline backbone alone (AC, FEC, FEC100), anthracycline–taxane backbone (ACD, ACT, FECD, FECT), other (paclitaxel, docetaxel, capecitabine, TC) and unknown. Abbreviations: T stage—tumour size; N stage—nodal status; M stage—metastasis; G1—Nottingham grade 1; G2—Nottingham grade 2; G3—Nottingham grade 3; SD—standard deviation; ER+—estrogen receptor-positive; PR+—progesterone receptor-positive; HER2+—human epidermal growth factor 2-positive; TNBC—triple-negative breast cancer; LVI—lymphovascular invasion; NA—not available. Table S3: Distribution of first distant metastatic sites over time. Sites of distant metastasis were classified as bone, brain and visceral (lung, liver, organs of the mediastinum). Abbreviations: DM—distant metastasis. Table S4: Average time in months to first distant metastasis for each site. Sites of distant metastasis were classified as bone, brain and visceral (lung, liver, organs of the mediastinum). Table S5: Frequency of metastatic sites and average times to metastasis according to subtype. Sites of distant metastasis were classified as bone, brain and visceral (lung, liver, organs of the mediastinum). Subtypes were grouped as follows: Luminal A (ER+, PR+, HER2-), Luminal B (ER+, PR+/−, HER2+), HER2 Enriched (ER-, PR-, HER2+), TNBC (ER-, PR-, HER2-).
