# Supplementary material for: Predicting Patterns of Distant Metastasis in Breast Cancer Patients following Local Regional Therapy Using Machine Learning
Source: Genes (Basel). 2023 Sep 7;14(9):1768. doi: 10.3390/genes14091768 (PMC10531341; doi:10.3390/genes14091768)
Supplement: Supplementary file 1 [file genes-14-01768-s001.zip › genes-2562962-supplementary.pdf]

Supplementary Materials:

Table S1. Clinicopathological characteristics of patient cohorts grouped by the site of first distant metastasis. Sites of distant metastasis were classified as bone, brain and visceral (lung, liver, organs of the mediastinum). Chemotherapy treatment was grouped as follows: Anthracycline backbone alone (AC, FEC, FEC100), anthracycline-taxane backbone (ACD, ACT, FECD, FECT), other (paclitaxel, docetaxel, capecitabine, TC) and unknown. Abbreviations: T stage, tumour size; N stage, nodal status; M stage, metastasis; G1, Nottingham grade 1; G2, Nottingham grade 2; G3, Nottingham grade 3; SD, standard deviation; ER+, estrogen receptor positive; PR+, progesterone receptor positive; HER2+, human epidermal growth factor positive; TNBC, triple-negative breast cancer; LVI, lymphovascular invasion; NA, not available.

Clinicopathological Characteristics	Bone Metastasis (n=99)	Brain Metastasis (n= 55)	Visceral Metastasis (n=21)
a) Pre-surgical			
Age			
Mean Age±SD years	56.23±13.62	54.02±12.35	56.52±14.84
20-39 years	9 (9%)	9 (16%)	3 (14%)
40-49 years	29 (29%)	13 (24%)	3 (14%)
50-59 years	22 (22%)	14 (25%)	7 (33%)
60-69 years	20 (20%)	16 (29%)	2 (10%)
≥70 years	19 (19%)	3 (6%)	6 (29%)
Laterality			
Left	47 (47%)	33 (60%)	13 (62%)
Right	52 (52%)	22 (40%)	8 (38%)
b) Surgical Pathology			
Surgical Procedure			
Lumpectomy	70 (71%)	36 (65%)	16 (76%)
Mastectomy	29 (29%)	19 (35%)	5 (24%)
T Stage			
Mean size±SD (mm)	32.30±18.41	32.24±19.21	25.98±19.51
T1	22 (22%)	14 (25%)	10 (48%)
T2	63 (64%)	33 (60%)	10 (48%)
T3	13 (13%)	7 (13%)	1 (5%)
T4	1 (1%)	1 (2%)	0 (0%)
N Stage			
N0	22 (22%)	21 (38%)	9 (43%)
N1	44 (44%)	14 (25%)	6 (29%)
N2	20 (20%)	13 (24%)	3 (14%)
N3	13 (13%)	7 (13%)	3 (14%)
Nottingham Grade			
G1	8 (8%)	1 (2%)	3 (14%)
G2	36 (36%)	15 (27%)	5 (24%)
G3	54 (55%)	39 (71%)	13 (62%)
NA	1(1%)	0 (0%)	0 (0%)
Receptor Status			
ER+	77 (78%)	23 (42%)	12 (57%)
PR+	76 (77%)	22 (40%)	11 (52%)
HER2+	13 (13%)	16 (29%)	5 (24%)

Subtype			
Luminal A	67 (38%)	16 (9%)	9 (5%)
Luminal B	10 (6%)	7 (4%)	3 (3%)
HER2 Enriched	3 (3%)	9 (5%)	2 (1%)
TNBC	19 (11%)	23 (13%)	7 (4%)
LVI Status			
LVI-	38 (38%)	30 (55%)	8 (38%)
LVI+	54 (53%)	20 (36%)	12 (57%)
NA	8 (8%)	5 (9%)	1 (5%)
c) Adjuvant Treatments			
Chemotherapy	n= 73 (74%)	n= 46 (84%)	n= 16 (76%)
Anthracycline backbone alone	6 (6%)	3 (5%)	0 (0%)
Anthracycline-Taxane backbone	56 (57%)	35 (64%)	10 (48%)
Other	2 (2%)	2 (4%)	6 (29%)
Unknown	9 (9%)	6 (11%)	0 (0%)
Endocrine Therapy	(n= 45 (45%))	n= 25 (45%)	n= 10 (47%)
Aromatase Inhibitors	29 (29%)	8 (16%)	6 (29%)
Tamoxifen	9 (9%)	12 (22%)	2 (10%)
Unknown	7 (7%)	5 (9%)	2 (10%)
Trastuzumab	34 (34%)	10 (18%)	4 (19%)

Table S2. Clinicopathological characteristics of patient cohorts grouped by the time in years until first distant metastasis. Chemotherapy treatment was grouped as follows: Anthracycline backbone alone (AC, FEC, FEC100), Anthracycline-Taxane backbone (ACD, ACT, FECD, FECT), other (paclitaxel, docetaxel, capecitabine, TC). and unknown. Abbreviations: T stage, tumour size; N stage, nodal status; M stage, metastasis; G1, Nottingham grade 1; G2, Nottingham grade 2; G3, Nottingham grade 3; SD, standard deviation; ER+, estrogen receptor-positive; PR+, progesterone receptor-positive; HER2+, human epidermal growth factor positive; TNBC, triple-negative breast cancer; LVI, lymphovascular invasion; NA, not available.

Clinicopathological Characteristics	≤1 year (n=22)	>1-≤2 years (n=40)	>2-≤3 years (n=33)	>3-≤4 years (n=21)	>4-≤5 years (n=22)	>5 years (n=37)
a) Pre-surgical						
Age						
Mean Age±SD years	58.50±16.49	52.98±13.80	59.09±12.90	56.33±10.22	55.27±11.54	53.24±13.53
<50 years	9 (41%)	19 (28%)	10 (30%)	5 (24%)	9 (42%)	14 (38%)
Laterality						
Left	13 (59%)	27 (68%)	9 (27%)	9 (43%)	13 (59%)	22 (59%)
Right	9 (41%)	13 (33%)	24 (73%)	12 (57%)	9 (41%)	15 (41.5%)
b) Surgical Pathology						
Surgical Procedure						
Lumpectomy	13 (59%)	27 (68%)	28 (85%)	13 (62%)	14 (64%)	27 (73%)
Mastectomy	9 (41%)	13 (33%)	5 (15%)	8 (38%)	8 (36%)	10 (27%)
T Stage						
Mean size±SD (mm)	32.33±14.87	26.95±28.92	28.39±14.98	24.43±11.66	38.18±24.97	27.93±17.66
T1	4 (18%)	5 (13%)	10 (30%)	8 (38%)	5 (23%)	14 (38%)
T2	15 (68%)	28 (70%)	22 (67%)	12 (57%)	12 (55%)	17 (46%)
T3	2 (9%)	7 (18%)	1 (3%%)	1 (5%)	4 (18%)	6 (16%)

T4	1 (5%)	0 (0%)	0 (0%)		1 (5%)	0 (0%)
N Stage						
N0	5 (23%)	9 (23%)	13 (39%)	3 (14%)	8 (36%)	14 (38%)
N1	7 (32)	18 (45%)	12 (36%)	12 (57%)	4 (18%)	11 (30%)
N2	5 (23%)	10 (25%)	5 (15%)	2 (10%)	5 (23%)	18 (49%)
N3	5 (23%)	3 (8%)	3 (9%)	4 (19%)	5 (23%)	3 (8%)
M Stage						
M0	22 (100%)	40 (100%)	33 (100%)	21 (100%)	22 (100%)	37 (100%)
Nottingham Grade						
G1	3 (14%)	0 (0%)	2 (6%)	1 (5%)	2 (9%)	4 (11%)
G2	4 (18%)	12 (30%)	6 (18%)	7 (33%)	11 (50%)	16 (43%)
G3	15 (68%)	28 (70%)	25 (76%)	12 (57%)	9 (41%)	17 (46%)
NA	0 (0%)	0 (0%)	0 (0%)	1 (5%)	0 (0%)	
Receptor Status						
ER+	6 (27%)	16 (40%)	21 (64%)	18 (86%)	20 (91%)	31 (84%)
PR+	6 (27%)	15 (38%)	21 (64%)	18 (86%)	19 (86%)	30 (81%)
HER2+	4 (18%)	9 (23%)	3 (9%)	3 (14%)	5 (23%)	10 (27%)
Subtype						
Luminal A	6 (27%)	12 (30%)	19 (58%)	16 (76%)	16 (73%)	23 (62%)
Luminal B	0 (0%)	4 (10%)	2 (6%)	2 (10%)	4 (18%)	8 (22%)
HER2 Enriched	4 (18%)	5 (13%)	1 (3%)	1 (4%)	1(4%)	2 (5%)
TNBC	12 (55%)	19 (23%)	11 (33%)	2 (10%)	1(4%)	4 (11%)
LVI Status						
LVI-	10 (45%)	22 (55%)	13 (39%)	7 (33)	9 (41%)	16 (43%)
LVI+	12 (55%)	15 (38%)	17 (52%)	12 (57%)	11 (50%)	17 (46%)
NA	0 (0%)	3 (8%)	3 (9%)	2 (10%)	2 (9%)	4 (11%)
c) Adjuvant Treatments						
Chemotherapy	N=16 (73%)	N= 32 (80%)	N= 23 (70%)	N= 18 (86%)	N= 15 (68%)	N= 31 (84%)
Anthracycline backbone alone	0 (0%)	2 (5%)	2 (6%)	1 (5%)	0 (0%)	4 (11%)
Anthracycline-Taxane backbone	14 (64%)	26 (65%)	16 (48%)	12 (57	11 (50%)	22 (59%)
Other	2 (9%)	1 (3%)	1 (3%)	3 (14%)	2 (9%)	1 (3%)
Unknown	0 (0%)	3 (8%)	4 (12%)	2 (10%)	2 (9%)	4 (11%)
Endocrine Therapy	N=10 (45%)	N=15 (38%)	N= 18 (55%)	N= 16 (76%)	N= 19 (86%)	N= 27 (73%)
Aromatase Inhibitors	4 (18%)	4 (1%)	7 (21%)	10 (48%)	5 (23%)	13 (35%)
Tamoxifen	3 (14%)	8 (20%)	8 (24%)	5 (24%)	11 (50%)	13 (35%)
Unknown	3 (14%)	3 (8%)	3 (9%)	1 (5%)	3 (14%)	1 (3%)
Trastuzumab	4 (18%)	8 (20%)	1 (3%)	2 (10%)	2 (9%)	6 (16%)

Table S3: Distribution of first distant metastatic site over time. Sites of distant metastasis were classified as bone, brain and visceral (lung, liver, organs of the mediastinum). Abbreviations: DM, distant metastasis.

Site of DM	≤1 year (n=22)	>1-≤2 years (n=40)	>2-≤3 years (n=33)	>3-≤4 years (n=21)	>4-≤5 years (n=22)	>5 years (n=37)
Bone	8 (36%)	21 (53%)	20 (61%)	17 (81%)	15 (68%)	18 (49%)
Brain	11 (50%)	13 (33%)	9 (27%)	3 (14%)	5 (23%)	14 (38%)
Visceral	3 (14%)	6 (15%)	4 (12%)	1 (5%)	2 (9%)	5 (14%)

Table S4: Average time in months to first distant metastasis for each site. Sites of distant metastasis were classified as bone, brain and visceral (lung, liver, organs of the mediastinum).

	Bone Metastasis (n=99)	Brain Metastasis (n= 55)	Visceral Metastasis (n=21)
Average Time to first DM (months)	41.92±25.48	41.51±35.61	36.44± 25.74
Median Time to first DM (months)	37.58	31.30	30.58

Table S5: Frequency of metastatic site and average time to metastasis according to subtype. Sites of distant metastasis were classified as bone, brain and visceral (lung, liver, organs of the mediastinum). Subtypes were grouped as follows: Luminal A (ER+, PR+, HER2-), Luminal B (ER+, PR+/-, HER2+), HER2 Enriched (ER-, PR-, HER2+), TNBC (ER-, PR-, HER2-).

Site of DM	Luminal A (n= 92)	Luminal B (n= 20)	HER2 Enriched (n= 14)	TNBC (n= 49)
Bone (% of subtype)	**67 (72%)**	**10 (50%)**	3 (21%)	19 (39%)
Brain (% of subtype)	16 (17%)	7 (35%)	**9 (64%)**	**23 (47%)**
Visceral (% of subtype)	9 (10%)	3 (15%)	2 (14%)	7 (14%)
Average Time to first DM (months)	47.77	54.53	30.33	26.30
